# Can prothrombotic gene variants and Apoa1 rs5069 polymorphism be the predictors of early myocardial infarctions?

**DOI:** 10.55730/1300-0144.5837

**Published:** 2024-06-12

**Authors:** Hüseyin BALCIOĞLU, Elif Fatma ÖZKAN PEHLİVANOĞLU, Uğur BİLGE, Kadir Uğur MERT, Muhammet DURAL, Ebru ERZURUMLUOĞLU GÖKALP, Oğuz ÇİLİNGİR, Sevilhan ARTAN

**Affiliations:** 1 Department of Family Medicine, Faculty of Medicine, Eskişehir Osmangazi University, Eskişehir, Turkiye; 2 Eskişehir Local Health Authority, Eskişehir, Turkiye; 3 Department of Cardiology, Faculty of Medicine, Eskişehir Osmangazi University, Eskişehir, Turkiye; 4 Department of Medical Genetics, Faculty of Medicine, Eskişehir Osmangazi University, Eskişehir, Turkiye

**Keywords:** Gene mutation, myocardial infarction at a young age, polymorphism

## Abstract

**Background/aim:**

We aimed to determine the genetic risk factors in patients aged 45 years and below with a history of early myocardial infarction (MI), compared to individuals over 60 years of age with no history of MI.

**Materials and methods:**

In this study, we selected different age groups to more clearly distinguish genetic differences. Accordingly, we compared individuals who had experienced MI at an early age with those who were older and had not experienced any cardiovascular events. The patient group consisted of 99 volunteers under the age of 45 with a history of MI, while the control group included 99 volunteers aged 60 and over without a history of MI. MTHFR (C677T, A1298C), Factor V Leiden (G1691A), Prothrombin (G20210A), PAI (4G/5G), Factor XIII (V34L), APOA1 (rs670, rs1799837, rs5069), and APOB were studied using blood samples taken from the patients.

**Results:**

In the logistic regression analysis of thrombophilia markers and gene polymorphisms in the patient and control groups, no statistically significant increase was observed in markers other than APOA1 rs5069 gene polymorphism. APOA1 rs5069 gene polymorphism was found to be higher in the patient group than those without this polymorphism. The frequencies of homozygous MTHFR (C677T, A1298C) and heterozygous Factor XIII V34L were higher in the patient cohort compared to the controls.

**Conclusion:**

In our study, we found that prothrombotic gene variants and APOA1 rs5069 polymorphism were statistically significantly associated with coronary artery disease. Thus, prothrombotic gene variants and APOA1 rs5069 polymorphism may serve as predictors of early myocardial infarctions. Individuals with early family histories of coronary artery disease could be screened for these mutations.

## 1. Introduction

The etiology of cardiovascular diseases is multifactorial. Investigating the molecular genetics of atherosclerosis, in addition to traditional risk factors, is important in determining genetic risk factors for cardiovascular diseases [1]. Patients who had a myocardial infarction (MI) at a young age, with a positive family history and unknown risk factors for the disease, have demonstrated the necessity of genetic research. Numerous epidemiological studies have shown that disorders related to plasma lipid levels are a significant issue in early coronary artery disease (CAD) [2].

Apolipoprotein (APO) A1 gene encodes a large protein component that belongs to high-density lipoprotein (HDL). This component primarily transports lipoproteins from peripheral tissues to the liver [3]. The APOB gene plays a critical role in lipid transport. Some variants of the APOB gene lead to changes in its structure and function, which can result in disorders of lipid metabolism, such as an increase in plasma low-density lipoprotein (LDL) increase or a decrease in HDL [4]. Although some studies have suggested that APOB (rs693) polymorphism is a risk factor for CAD in recent years, there is not enough data on this subject [5]. It is predicted that APOA1 gene rs670 and rs5069 polymorphisms may cause heart disease by raising systolic blood pressure and plasma glucose levels [6]. There is some evidence that APOA1 rs1799837 polymorphism may be associated with heart disease by causing a decrease in HDL levels [3].

There is no consensus on whether prothrombotic gene variants are risk factors for arterial thrombosis such as acute MI [7,8]. Current sources recommend investigating activated protein C resistance, Factor V Leiden and prothrombin gene G20210A mutation, protein S and antithrombin activities as a panel for hereditary thrombophilia [9]. This study aims to investigate the effects of MTHFR (C677T, A1298C), Factor V Leiden (G1691A), Prothrombin (G20210A), PAI (4G/5G) and Factor XIII (V34L), APOA1 (rs670, rs1799837, rs5069) and APOB rs693 polymorphisms in young patients who have experienced MI. Specifically, we aim to investigate the relationship between prothrombotic gene variants, APOA1 rs5069 polymorphism, and early myocardial infarction.

## 2. Materials and method

### 2.1. Participants

The study was conducted in accordance with the guidelines of the Declaration of Helsinki. Ethical approval was received from the Eskişehir Osmangazi University Non-Interventional Clinical Research Ethics Committee on March 06, 2017, with reference number 80558721/G-85 and decision number 12. Patients were recruited from the Cardiology and Family Medicine Clinics at Eskişehir Osmangazi University Faculty of Medicine Hospital. All samples were collected after obtaining written informed consent.

In this study, we selected different age groups to more accurately distinguish genetic differences. Specifically, we compared individuals who had experienced MI at an early age with those who were older and had not experienced any cardiovascular events. We conducted a study on a group of 99 patients, all under the age of 45, who had a history of myocardial infarction (MI). Additionally, we included a control group of 99 volunteers aged 60 and above who visited the cardiology or family medicine outpatient clinics but did not have a history of MI.

### 2.2. Genetic analysis

Genomic DNA was isolated from peripheral lymphocytes using PureLink Genomic DNA Mini Kit (Invitrogen Life Technologies, Carlsbad, CA, USA), following the manufacturer’s recommendations. To analyze MTHFR (C677T, A1298C), Factor V Leiden (G1691A), Prothrombin (G20210A), PAI (4G/5G), and Factor XIII (V34L) variants, we initially employed SNP analysis of these regions based on multiplex PCR, using GML SNP detective thrombophilia kit (Genomed, Altendorf, Switzerland) according to the manufacturer’s instructions. The multiplex system contains six primer pairs for target amplification and twelve single-base-specific DNA probes for SNP detection.

In addition, APOA1 (rs670, rs1799837, rs5069) and APOB rs693 variants were investigated using SNaPshot technique. This technique depends on PCR amplification followed by primer extension. The primer sequences for these analyses are as follows: APOA1-F (5′-GGGATGAGTGCAGGGAACCC-3′), APOA1-R(5′-GGTGGGCCACGGGGATTTAG-3′), APOB-F (5′-GTATCTGGAAAGCCTACAGGACACC-3′), APOB-R(5′-ACTTCRAAGGCAGGCATGGTCC-3′), rs5069-F(5′-GCCTTGCCCCAGGC-3′), rs670-R(5′-TTGCTGATAAGCCCAGCCC-3′), rs1799837-R(5′-TTTTTTTCTCAGGTACCCAGAGGCC-3′), and rs693-F(5′-TTTTTTCATGAAGGCCAAATTCCGAGAGAC-3′).Electrophoresis of amplified PCR products was performed using the ABI 3130 Genetic Analyzer, and all data were analyzed with the GeneMapper 4.0 Software (Applied Biosystems, Life Technologies, Foster City, CA, USA).

### 2.3. Statistical analysis

In statistical analysis, continuous data are presented as mean ± standard deviation, while categorical data are expressed as percentages (%). Pearson chi-square, Yates chi-square, Pearson exact chi-square, and Fisher exact chi-square analyses were used to analyze cross tables. Logistic regression analysis was employed for risk analysis. IBM SPSS Statistics v22.0 was used to perform the analyses. Statistically significant evidence of association was determined by p values of 0.05 or below.

## 3. Results

### 3.1. Demographic and clinical features of the study groups

A total of 198 participants, including 99 in the patient group and 99 in the control group, were included in our study. In the patient group, 19.19% (n = 19) were female and 80.80% were male. In the control group, 29.3% (n = 29) were female and 70.7% (n = 70) was male. The average age of the patient group was 39.17 ± 4.41 years, while the average age of the control group was 67.60 ± 6.98 years. The distributions of chronic diseases and smoking status of the participants are presented in Table 1.

### 3.2. Results of genetic analysis

The frequencies of homozygous MTHFR (C677T, A1298C) and heterozygous Factor XIII V34L were higher in the patient cohort compared to the controls. The difference in frequencies between the cases and controls was statistically significant (Table 2). The distribution of the thrombophilia panel among the participants was also displayed in Figure.

In the interpretation of thrombophilia markers and gene polymorphisms using logistic regression analysis in the patient and control groups, no statistically significant risk increase was observed for markers other than APOA1 rs5069 gene polymorphism. As presented in Table 3, individuals with APOA1 rs5069 gene polymorphism were 9.8-fold more likely to have MI than those without this polymorphism.

No statistically significant difference was found in the risk analysis for myocardial infarction between thrombophilia markers and gene polymorphisms in individuals without DM or HT (p > 0.05).

## 4. Discussion

Although acute coronary syndrome (ACS) is relatively rare in young adults compared to older adults, it is characterized by different clinical findings and is almost always difficult to diagnose due to its broader range of etiologies [10,11]. The idea that young people are protected against ACS, which is considered a disease of the elderly, is gradually being abandoned [12]. Potential causes of early MI include a stressful working environment, excessive workload, sedentary lifestyle, unhealthy eating habits, smoking, and addiction [13]. However, genetic factors also play an important role in cardiovascular events. For this reason, research has intensified to identify the genetic variations that can be used in diagnosis [14].

Prothrombotic gene variants provide inhibition of hemostasis and cause procoagulant effects [15]. These gene variants may cause recurrent venous thrombosis. However, there is no consensus on evaluating prothrombotic gene variants as risk factors for arterial disorders, including MI at a young age [16]. In our study, the relationship between prothrombotic gene variants and MI status was examined. Homozygous mutations of MTHFR A1298C and MTHFR C677T and heterozygous mutations of Factor XIII gene were statistically significantly higher in the patient group than in the control group (p = 0.009, p = 0.015, p = 0.014). Samii et al. focused on the relationship between MTHFR gene polymorphisms (C677T and A1298C) and MI. They observed a noteworthy association across different genotype models [17]. Golestani et al. studied association between thrombophilia and MI and indicated that MTHFR C677T polymorphism could contribute to susceptibility to MI and increase creatinine levels in the population of East Iran [18]. Mallhi T et al. showed in 2023 that there was a high correlation between MTHFR polymorphism and MI in the presence of diabetes mellitus as a risk factor. They emphasized that this correlation may also be related to genetic predisposition [19]. Identifying other diseases that may affect the high level of relationship may contribute to elucidating the genetic background. We consider whether prothrombotic gene variants contribute to the etiology of MI, and it would be useful to evaluate this with additional studies.

Al-Bustan et al. studied the relationship between polymorphisms of the APOA1 gene and high cholesterol levels and found that individuals with APOA1 rs670 polymorphism had high LDL cholesterol [3]. LDL cholesterol elevation is considered a risk factor for CAD. In our study, the role of APOA1 rs670 polymorphism in the etiology of cases of MI in young individuals was examined, and no significant relationship between MI status and polymorphism was found (p = 0.242).

The APOA1 rs5069 allele was associated with high HDL cholesterol and low triglyceride levels, while the APOA1 rs1799837 allele was associated with HDL cholesterol level and heart disease [20]. Wang et al. examined the relationship between the polymorphisms rs5069, rs5072, rs7116797, rs2070665, and rs1799837 of the APOA1 gene and found no significant relationship between them [21]. Sohail H et al. examined the relationship between APOA1 and B gene polymorphisms and CAD in Pakistan in 2024. In their study, they reported that the frequency of APOA rs5069 TT genotype and T allele (OR = 0.53, p = 0.35) increased in CAD, but this relationship was not significant [22]. We found no significant relationship between APOA1 rs1799837 polymorphism and MI status (p = 0.081). However, rs5069 heterozygous polymorphism was significantly higher in young MI cases than in healthy adults (p = 0.001). Additionally, individuals with APOA1 rs5069 polymorphism had a 9.8-fold higher risk of MI than those without (OR = 9.800; 95% CI=1.217–78.893; p = 0.032). Due to these differences among current studies, the effect of APOA1 rs5069 gene polymorphism on the etiology of CAD, either alone or in combination with environmental factors, should be revealed in studies involving large populations. We suggest that the studies on APOA1 rs5069 polymorphism may be useful in explaining the etiology of MI.

The main apolipoproteins of chylomicrons and low-density lipoproteins (LDL) are encoded by APOB. Hypobetalipoproteinemia, hypercholesterolemia and normotriglyceridemic hypobetalipoproteinemia are caused by mutations in the APOB or its regulatory region [23]. The relationship between APOB XbaI polymorphism and CAD risk has been investigated in several case-control studies conducted in different countries. However, the results of these studies have not been consistent [5].

In a meta-analysis including 12 articles with a total of 1596 individuals diagnosed with CAD and 1431 individuals in the control group, no significant relationship was found between Apolipoprotein B gene polymorphism and CAD risk [24]. Our study similarly found no significant relationship between MI and APOB XbaI polymorphism. With the development of new and comprehensive genetic studies, we think that the genetic structure of MI will become explainable.

The limitations of our study were that only eligible patients were included and the lack of consideration of other specific genetic factors.

## 5. Conclusion

In our study, we found that prothrombotic gene variants and APOA1 rs5069 polymorphism were statistically significantly associated with CAD. Additionally, for a complex disease such as CAD, which is considered the result of complex interactions between multiple genes and gene-environment interactions, expanded research is needed to identify APOA1 rs5069 and functional interacting genes and environmental triggers. In conclusion, we found that prothrombotic gene variants and APOA1 rs5069 polymorphism were statistically significantly associated with coronary artery disease. Therefore, prothrombotic gene variants and APOA1 rs5069 polymorphism could serve as predictors of early myocardial infarctions, and individuals with early family histories could be screened for these mutations.

## Figures and Tables

**Figure:**
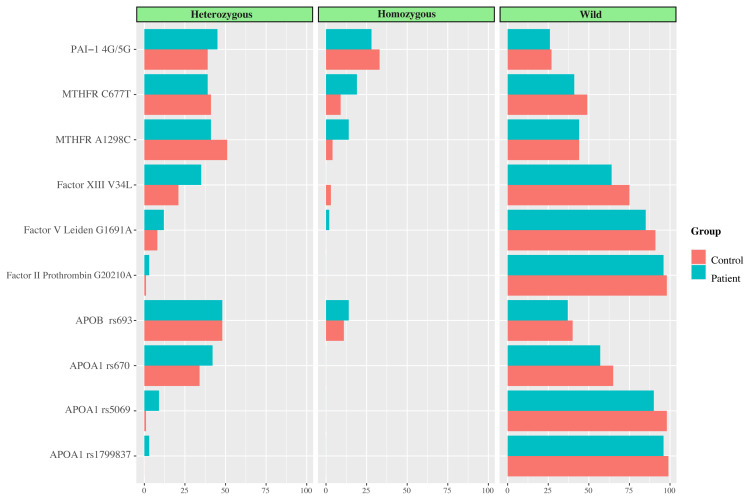
Thrombophilia panel distribution of participants.

**Table 1:** Distribution of chronic disease history and smoking status of the participants.

Chronic diseases	Patient group (n = 99)	Control group (n = 99)	p value
**Diabetes mellitus (DM)**	12 (12.12%)	0 (0.00%)	0.001
**Hypertension (HT)**	11 (11.10%)	0 (0.00%)	0.002
**Hyperlipidemia**	21 (21.21%)	0 (0.00%)	<0.001
**Smoking**	85 (85.85%)	11 (11.00%)	<0.001

**Table 2:** The thrombophilia panel and APOA and APOB gene polymorphisms of the participants.

The thrombophilia panel and gene polymorphisms		Group				p value
		Control (n = 99)		Patient (n = 99)		
		n	%	n	%	
***MTHFR*** ** C677T**	Wild	49	49.5%	41	41.4%	0.297
	Heterozygous	41	41.4%	39	39.4%	0.874
	Homozygous	9	9.0%	19	19.2%	**0.015**
***MTHFR*** ** A1298C**	Wild	44	44.4%	44	44.4%	1.000
	Heterozygous	51	51.5%	41	41.4%	0.184
	Homozygous	4	4.0%	14	14.1%	**0.009**
***Factor II Prothrombin*** ** G20210A**	Wild	98	99.0%	96	97.0%	0.919
	Heterozygous	1	1.0%	3	3.0%	0.486
	Homozygous	0	0.0%	0	0.0%	
***Factor V Leiden*** ** G1691A**	Wild	91	91.9%	85	85.9%	0.594
	Heterozygous	8	8.1%	12	12.1%	0.343
	Homozygous	0	0.0%	2	2.0%	
***Factor XIII*** ** V34L**	Wild	75	75.8%	64	64.6%	0.230
	Heterozygous	21	21.2%	35	35.4%	**0.014**
	Homozygous	3	3.0%	0	0.0%	
***PAI-1*** ** 4G/5G**	Wild	27	27.3%	26	26.3%	1.000
	Heterozygous	39	39.4%	45	45.5%	0.441
	Homozygous	33	33.3%	28	28.3%	0.469
***APOA1*** ** rs670**	Wild	65	65.7%	57	57.6%	0.370
	Heterozygous	34	34.3%	42	42.4%	0.256
	Homozygous	0	0.0%	0	0.0%	
***APOA1*** ** rs5069**	Wild	98	99.0%	90	90.9%	0.470
	Heterozygous	1	1.0%	9	9.0%	**0.001**
	Homozygous	0	0.0%	0	0.0%	
***APOA1*** ** rs1799837**	Wild	99	100.0%	96	97.0%	0.840
	Heterozygous	0	0.0%	3	3.0%	
	Homozygous	0	0.0%	0	0.0%	
***APOB*** ** rs693**	Wild	40	40.4%	37	37.4%	0.747
	Heterozygous	48	48.5%	48	48.5%	1.000
	Homozygous	11	11.1%	14	14.1%	0.572

**Table 3:** Logistic regression analysis of APOA1 rs5069 gene polymorphism in patient and control groups.

	B	SE	p	OR	95% CL	
					Lower	Upper
***APOA1*** **rs5069**	2.282	1.064	0.032	9.800	1.217	78.893
